# Extremely Rare Intraoral Presentation of Localized Amyloidosis

**DOI:** 10.1155/2021/5541320

**Published:** 2021-07-23

**Authors:** Luka Morelato, Igor Smojver, Sven Seiwerth, Dragana Gabrić

**Affiliations:** ^1^Department of Oral Surgery, Faculty of Dental Medicine, University of Rijeka, Croatia; ^2^Special Hospital, St. Catherine, Zagreb, Croatia; ^3^Department of Pathology, School of Medicine, University of Zagreb, Croatia; ^4^Department of Oral Surgery, School of Dental Medicine, University of Zagreb, Croatia

## Abstract

**Purpose:**

To present an extremely rare case of localized amyloidosis of the mucosa of the maxillary alveolar ridge. *Case Report*. A 71-year-old man was referred to the Department of Oral Surgery, School of Dental Medicine, University of Zagreb, for a persistent nodular formation in the edentulous ridge of the anterior maxillary region. The nodular formation had a reddish color, solid consistency, and an approximate size of 40 × 15 mm. Orthopantomographic imaging excluded bone resorption and defects. Histopathological assessments of the biopsy specimen showed that the stroma was occupied by a multiplied, partially hyalinized connective tissue. The samples were subsequently stained with Congo red, and collagen accumulation under polarized light showed an apple-green birefringence indicating amyloid. Subsequently, the nodular formation was completely excised and a maxillary total denture was made. The patient showed normal tissue healing with no sign of recurrence at a follow-up assessment 1.5 years after the procedure.

**Conclusion:**

This is only the third reported case of localized amyloidosis of the alveolar ridge mucosa. Histopathological analysis is the first step for diagnosis, but systemic tests, blood counts, urinalysis, bone marrow biopsy, electrocardiography, and digestive endoscopy are recommended to rule out a systemic disease.

## 1. Introduction

The term “amyloidosis” is used to describe a progressive metabolic disease with abnormal amyloid protein deposits in one or more organs [1, 2]. To date, 36 human proteins have been shown to possess the potential to form extracellular amyloid aggregates that precipitate in the extracellular spaces of different organ tissues, causing functional damage. Of these, 14 proteins are associated with systemic amyloidosis and 19 are known to cause localized forms of the disease, whereas the remaining three cause both localized and systemic forms of amyloidosis [2, 3]. These different types of proteins are currently named after the specific protein that is precipitated, using the prefix “A” for “amyloid,” followed by the letters indicating the protein type.

Amyloidosis may be acquired or hereditary, and the disease presentation may be primary or secondary. It may be restricted to a single organ (localized form), or it may affect many organs (systemic form) [4]. The cause of primary amyloidosis is unknown, and it has not yet been linked to any primary disease. Monoclonal immunoglobulin amyloid is deposited in organs such as the liver, heart, kidney, and spleen, leading to systemic manifestations such as congestive heart failure and unexplained proteinuria [5]. In patients with systemic forms of amyloidosis, the prognosis depends on the type and stage of the disease in the affected organs [6]. Secondary amyloidosis is the most common form of systemic amyloidosis, accounting for approximately 45% of the cases of systemic amyloidosis [7]. It occurs secondarily to a series of primary diseases involving chronic inflammation, such as rheumatoid arthritis, sarcoidosis, Crohn's disease, ulcerative colitis, and tuberculosis. Chronic oral periodontitis has also been suggested to lead to secondary amyloidosis [8]. Proper periodontal treatment can effectively reduce the formation of inflammatory mediators caused by amyloidosis [9]. Intraoral amyloidosis is a rare and usually benign condition. The most commonly affected areas in the oral cavity are the tongue and buccal mucosa, and the involvement of other areas is considered to be extremely rare [10].

Localized amyloidosis is a rare type of disease characterized by the deposition of amyloid in the localized tissue of a particular anatomical region. It manifests as a tissue mass that can mimic a growing tumor [11]. If head and neck structures are involved, localized amyloidosis is usually a benign condition with a good prognosis [7]. The incidence of amyloidosis is approximately nine to twelve cases per million people per year [6, 12]. The diagnosis of amyloidosis is not difficult, since the lesions of the localized forms are mostly superficial and easy to observe and require a biopsy. Congo red staining is the gold standard for histological confirmation; it is used with polarized light microscopy under which amyloid deposits show a characteristic apple-green birefringence [12].

The aim of the present study was to report a rare case of intraoral localized amyloidosis discovered as an incidental finding due to the patient's inability to wear a maxillary total denture.

## 2. Case Presentation

**Figure 1:**
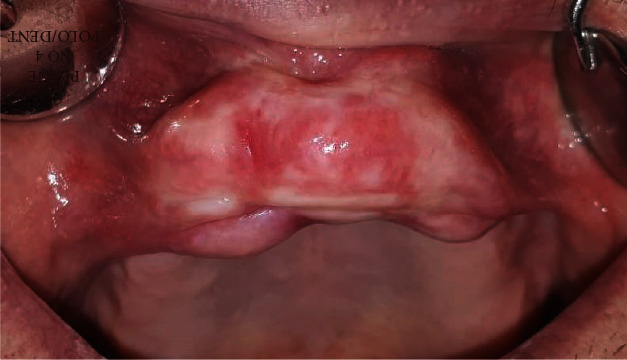
Slow-growing painless mass.

**Figure 2:**
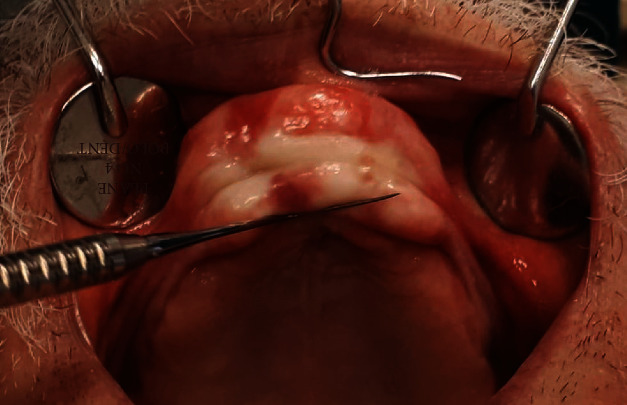
Intraoral findings.

A 71-year-old man was referred by his general dentist to the Department of Oral Surgery, School of Dental Medicine, University of Zagreb, in July 2019 because of a persistent nodular formation in the vestibular intercanine region of the edentulous ridge of the maxilla. The nodular formation was reddish-pink in color, had a solid consistency, and measured approximately 40 × 15 mm with a periodontal probe (Deppeler SA, Rolle, Switzerland) (Figure 1). The patient reported noticing this small and painless mass growing for the first time approximately 15 years ago, after his general dentist made a maxillary total denture. After time, denture was adapted to avoid the nodular mass. He had been wearing the total denture using a denture adhesive (Figure 2).

The patient had a history of type II diabetes mellitus and arterial hypertension. He had also undergone several medical procedures, including penile amputation due to planocellular carcinoma T2NxMx in February 2018, and lymphadenectomy in May of the same year. In August 2018, he broke his femoral neck and underwent left hip total endoprosthesis replacement. He also had cardiovascular disease in the form of aortic stenosis and ischemic cardiomyopathy and had undergone surgery for aortic valve replacement with a biological prosthesis (Dokimos 23 mm, Labcor, Calafate, Belo Horizonte) and a double coronary bypass in June 2019. He was regularly taking nebivolol 2.5 mg, trimetazidine 2 × 35 mg, indapamide 1.5 mg, acetylsalicylic acid 100 mg, ramipril 1.25 mg, atorvastatin 40 mg, and gliclazide 60 mg.

**Figure 3:**
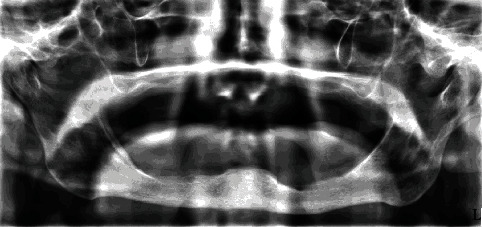
Orthopantomographic image showing no underlying bone resorption or defect.

After recovering from the cardiac surgery, the patient decided to get a new maxillary total denture when the nodular formation was observed. The preoperative differential diagnosis concluded a suspect epulis fissuratum due to irritation caused by a worn upper dental prosthesis or a growing neoplasm. Orthopantomographic imaging was performed, and underlying bone resorption and defects were excluded (Figure 3). Extraoral examination did not show enlarged lymph nodes in the head and neck area.

**Figure 4:**
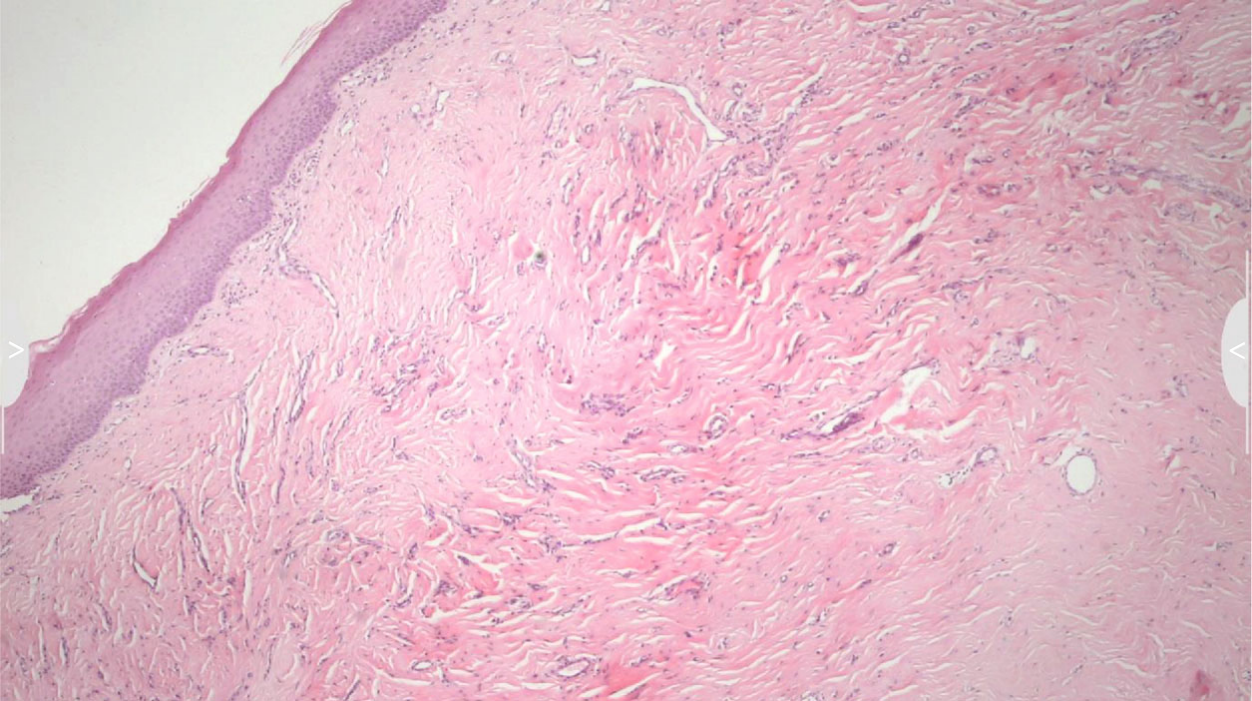
Slightly mottled, multilayered squamous epithelium over a stroma occupied by a multiplied, partially hyalinized binder.

**Figure 5:**
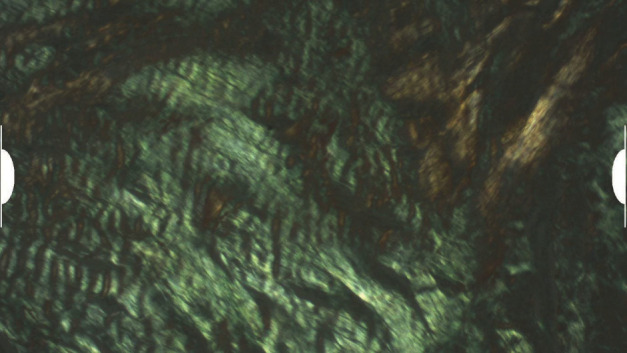
Collagen accumulations showing apple-green birefringence of amyloid under polarized light.

Consistent with the ethical requirements of the School of Dental Medicine, University of Zagreb, Croatia, written informed consent for the publication of this study was obtained from the patient prior to the surgery. In the first stage, biopsy material was obtained under local anesthesia (1.7 mL articaine 4%; 1 : 100000) by performing wedge incisions along the long side of the nodule formation; two tissue samples obtained with this procedure were sent for histopathological assessment. Excisional biopsy findings were analyzed after fixation in 6% buffered formalin, dehydration, and paraffin embedding of the tissue samples, after which 4 *μ*m thick sections were cut and stained with hematoxylin-eosin for diagnostic purposes. The histopathological assessments revealed a slightly mottled, multilayered squamous epithelium over a stroma occupied by a multiplied, partially hyalinized binder (Figure 4). The samples were subsequently stained with Congo red, following which collagen accumulations were observed with the apple-green birefringence of amyloid under polarized light (Figure 5). The histopathological findings indicated a case of amyloidosis.

Laboratory tests for blood and urine showed that proteinuria was absent and the complete blood count was normal. Lung radiography and brain computed tomography did not reveal any pathology. Electrocardiogram monitoring also showed normal findings after aortic valve replacement.

In the second stage of the procedure, after the application of local anesthesia (1.7 mL of articaine 4%; 1 : 100000), the formation was completely excised using electrocauterization (ART Electrosurge, Bonart Co., New Taipei City, Taiwan) with a wire electrode.

After a healing period of two weeks, a new, complete upper denture was made.

**Figure 6:**
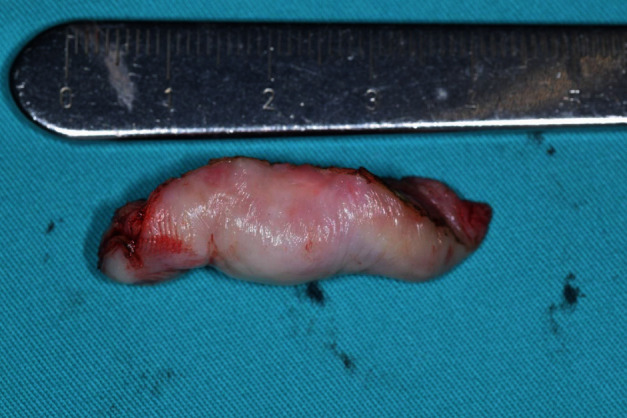
Ductile formation specimen after surgical excision.

The whole ductile formation measuring 3.2 × 1.3 × 1.1 cm was submitted for histopathological analysis, and the diagnosis of amyloidosis was confirmed (Figure 6).

**Figure 7:**
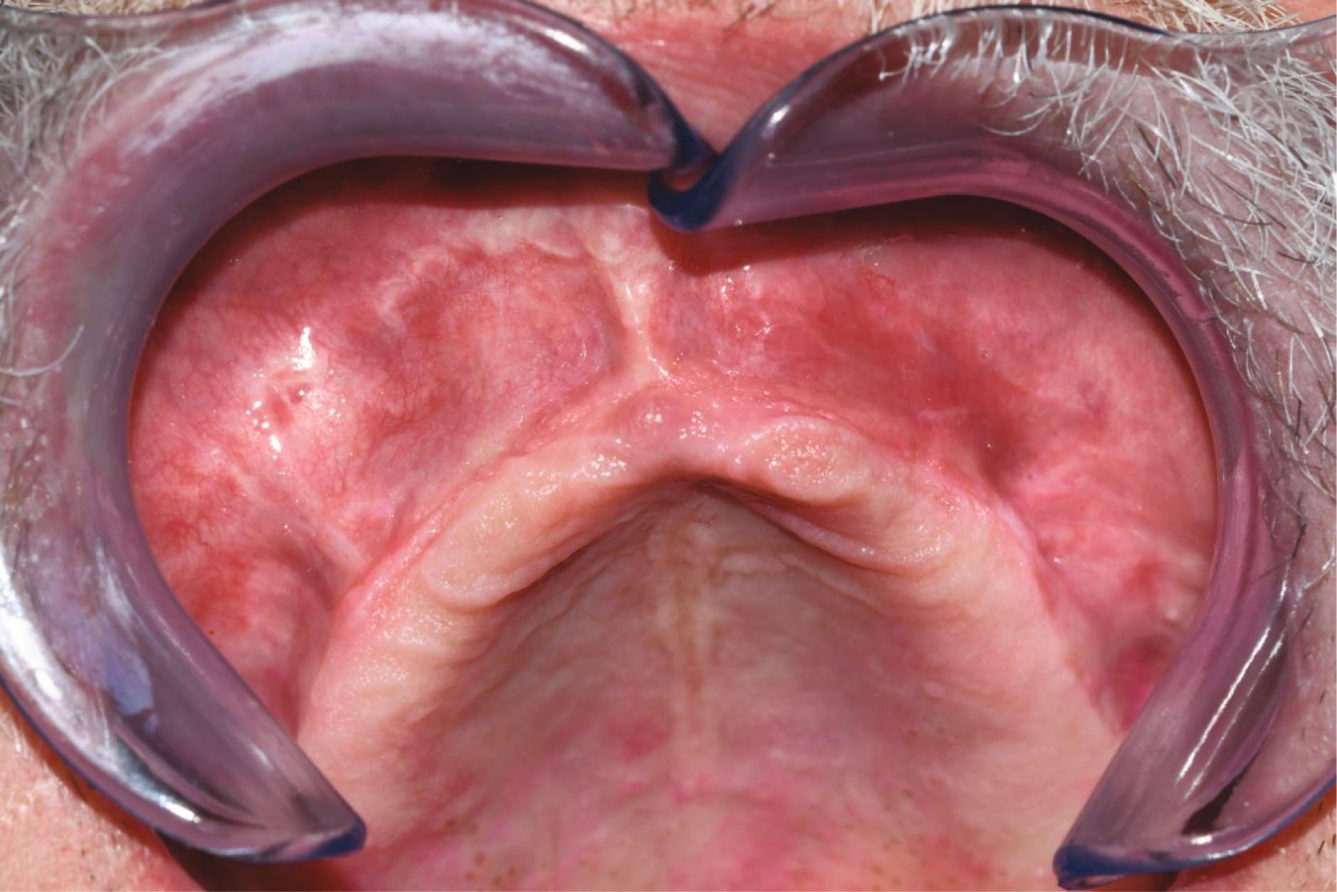
Normal tissue healing at the follow-up assessment after 1.5 years.

Based on the normal findings of all tests such as the blood and urine assessments, electrocardiography, gastrointestinal endoscopy, lung radiography, and brain computed tomography as well as the long and slow growth period of the nodular formation, a localized amyloidosis of the mucosa of the upper alveolar ridge was diagnosed. However, due to the patient's age and medical condition, he refused to undergo bone marrow biopsy to completely rule out systemic disease. The patient was followed up for 1.5 years, during which he showed normal tissue healing and no sign of recurrence (Figure 7).

## 3. Discussion

Localized intraoral amyloidosis is an extremely rare disease. The most commonly affected areas in the head and neck region are the larynx, thyroid, and subglottis. In the oral cavity, usually affected is the tongue, whereas the buccal mucosa is affected less often [13]. Amyloidosis in other locations is extremely rare. To the best of our knowledge, this is the third case of localized amyloidosis of the maxillary alveolar mucosa ever reported in the literature [14].

The etiology of localized amyloidosis itself is not completely clear; the first step in the development could be the reaction of plasma cells to environmental antigens and chronic inflammation, followed by abundant production of light chains of immunoglobulins that transform into insoluble fibrils and locally deposit in tissues [15]. Nevertheless, only a few plasma cells could be identified in the biopsy material, leading to the “suicidal neoplasm” theory [16].

In the present case, the chronic local irritation could be caused by an improperly adjusted upper removable prosthesis. The same was suggested in a second case reported in the literature by Bucci et al. [14, 17].

During the period of 15 years, painless mass was slowly growing without bleeding, ulceration, or acute inflammation periods. Due to the localization and retarded growth of the nodular formation during the total denture-wearing period, the differential diagnosis indicated epulis fissuratum, which typically occurs in the area of chronic irritation caused by inadequately adjusted denture margin [18] or a suspected malignant neoplasm due to the nodular mass. The patient has several severe illnesses considering that the literature could not be related to oral amyloidosis and by conducting tests that excluded potential organ involvement in systemic amyloidosis form [6].

Treatment of localized amyloidosis depends on the affected organ as well as on the degree of functional impairment [19, 20]. Surgical excision of the amyloid deposits has been suggested to avoid functional damage as a result of growth formation, although frequent recurrences have been described. However, there is no consensus on the optimal treatment approach, and there are no relevant surgical guidelines. No randomized controlled trials have been conducted, and the current knowledge is based mainly on the findings from a few case reports [21, 22]. In the literature, surgical excision was reported in nine cases, in which recurrence occurred in two cases. Namely, Babburi et al. performed complete surgical excision in a case of nodular tongue amyloidosis, which recurred 3 years later [23], and O'Reilly et al. reported recurrence in one case after a year, necessitating repeated excision [24]. Bleeding may be a major complication of the excision of localized amyloidosis, and it is explained by the lack of factor X and the fragility of blood vessels as a result of the interaction of blood with amyloid fibrils [18, 25]. The prognosis of localized oral amyloidosis is good. In previous retrospective studies, many authors did not report the transition of the disease from an oral localized to a systemic form [23, 26, 27]. In the present case, the patient was normally wearing the upper dental prosthesis made after the surgical excision of the lesion and showed normal tissue healing with no recurrence after 1.5 years.

Our findings are very similar to two previous cases reported in the literature, and it could be suggested that local amyloidosis of the alveolar mucosa could be caused by chronic irritation of ill-fitting dentures, but other studies on larger numbers of cases have to be conducted to confirm the finding.

According to mostly very clear clinical findings, with specific anamnesis in everyday practice, not all epulis fissuratum after excision were pathohistologically analyzed. This case shows unusual findings and suggests that all even typical specimens should sustain pathohistological diagnosis and according to that should be followed up properly.

In conclusion, our findings emphasize the need for cooperation among dentists, oral and maxillofacial surgeons, pathologists, and general practitioners in the diagnosis, treatment, and lifelong follow-up of patients diagnosed with local amyloidosis. After an excisional biopsy of the lesion, histological examination is the first step toward diagnosis, followed by the exclusion of the systemic form of the disease, and immunohistochemical tests are desirable. The diagnosis of localized amyloidosis should always be accompanied by blood tests, bone marrow biopsy, echocardiography, and gastrointestinal endoscopy to rule out systemic amyloidosis or any other hematological/immune disorders or organ dysfunctions. There is no consensus on the treatment of local amyloidosis. Surgical treatment is indicated to reduce the functional damage produced by the bulky mass. In any case, lifelong follow-up of the patient is recommended.

## Data Availability

The data generated during and/or analyzed during the current study are available from the corresponding author on reasonable request.
